# A novel solid state fermentation coupled with gas stripping enhancing the sweet sorghum stalk conversion performance for bioethanol

**DOI:** 10.1186/1754-6834-7-53

**Published:** 2014-04-08

**Authors:** Hong-Zhang Chen, Zhi-Hua Liu, Shu-Hua Dai

**Affiliations:** 1 State Key Laboratory of Biochemical Engineering, Institute of Process Engineering, Chinese Academy of Sciences, Beijing 100190, China; 2 Graduate University of Chinese Academy of Sciences, Beijing 100190, China

**Keywords:** Gas stripping (GS), Solid state fermentation (SSF), Traditional static solid state fermentation (TS-SSF), Sweet sorghum stalk (SSS), Bioethanol, Particle thickness, Ethanol stripping efficiency, Carbon dioxide weight loss

## Abstract

**Background:**

Bioethanol production from biomass is becoming a hot topic internationally. Traditional static solid state fermentation (TS-SSF) for bioethanol production is similar to the traditional method of intermittent operation. The main problems of its large-scale intensive production are the low efficiency of mass and heat transfer and the high ethanol inhibition effect. In order to achieve continuous production and high conversion efficiency, gas stripping solid state fermentation (GS-SSF) for bioethanol production from sweet sorghum stalk (SSS) was systematically investigated in the present study.

**Results:**

TS-SSF and GS-SSF were conducted and evaluated based on different SSS particle thicknesses under identical conditions. The ethanol yield reached 22.7 g/100 g dry SSS during GS-SSF, which was obviously higher than that during TS-SSF. The optimal initial gas stripping time, gas stripping temperature, fermentation time, and particle thickness of GS-SSF were 10 h, 35°C, 28 h, and 0.15 cm, respectively, and the corresponding ethanol stripping efficiency was 77.5%. The ethanol yield apparently increased by 30% with the particle thickness decreasing from 0.4 cm to 0.05 cm during GS-SSF. Meanwhile, the ethanol yield increased by 6% to 10% during GS-SSF compared with that during TS-SSF under the same particle thickness. The results revealed that gas stripping removed the ethanol inhibition effect and improved the mass and heat transfer efficiency, and hence strongly enhanced the solid state fermentation (SSF) performance of SSS. GS-SSF also eliminated the need for separate reactors and further simplified the bioethanol production process from SSS. As a result, a continuous conversion process of SSS and online separation of bioethanol were achieved by GS-SSF.

**Conclusions:**

SSF coupled with gas stripping meet the requirements of high yield and efficient industrial bioethanol production. It should be a novel bioconversion process for bioethanol production from SSS biomass.

## Background

The production of biofuels (largely bioethanol) from biomass has attracted much interest from governments around the world because of its higher octane number and higher heat of vaporization [1-4]. Agriculture straw biomass is the most abundant renewable resource on earth, and the annual yield is approximately 700 million tons in China [5,6]. Of the many agriculture straws currently being investigated for energy and industry, sweet sorghum stalk (SSS) is considered as a cost-effective feedstock for bioethanol production due to its higher drought resistant ability, lower production costs, and higher biomass yield (20 to 30 dry tons/ha) compared with other straws [7-9]. SSS, which is a C4 plant, can efficiently convert sunlight into stored chemical energy by photosynthetic fixation of atmospheric CO_2_ to produce sugars [7]. A large proportion of these sugars are stored either as soluble sugars or plant cell wall polymers, and hence SSS contains plentiful soluble carbohydrates (especially glucose, fructose, and sucrose) and insoluble carbohydrates (cellulose and hemicellulose), which can be converted into biofuels by microorganisms [8,9]. Meanwhile, sweet sorghum juice accounts for a large part of SSS biomass, which not only contains abundant soluble sugars used directly as a substrate for bioethanol production, but also provides efficient nutrient supplementation for microbe fermentation [8,10]. Therefore, SSS biomass should be the first competitor among the biological energy agriculture crops, and utilization of SSS for bioethanol production should be an effective way to reduce the process capital cost [11-14].

Previous literature has reported on bioethanol production using submerged fermentation (SmF) from sweet sorghum juice [15,16]. The juicing process of sweet sorghum juice from SSS biomass makes the bioconversion process tedious, and hence increases the capital cost of bioethanol production. Meanwhile, there are many shortcomings of the SmF process for biofuel production from solid substrate biomass, including high pretreatment costs, relatively low volumetric productivity, hard separation and purification of products, and much effluent generation [17-19]. Generally speaking, solid state fermentation (SSF), which is defined as a microbial culture that develops on natural solid substrates or impregnated inert supports in or near the absence of free water, offers numerous advantages (such as lower energy requirements, producing lesser wastewater, environmentalfriendly, and so on) over SmF, particularly with the possibility of using solid agro-industrial residues for biofuel production [20-22]. However, few reports are available using SSF from SSS biomass for bioethanol production. A summary of literature reports on SSF of SSS for bioethanol production is shown in Table 1 [23-25]. The traditional SSF for bioethanol production from SSS biomass is similar to the traditional method of intermittent operation. However, it does not meet the requirements of large-scale intensive production of bioethanol from solid substrate biomass. During the traditional SSF process, the absolute ethanol content in solid substrate is much higher than that during SmF, and the ethanol inhibition effect on yeast cells becomes more significant [4,26]. Since the thermal conductivity of solid substrates is very low and there is little free water in the reaction system, the removal of excess heat and the transfer of mass are other major problems for traditional SSF [18,20]. Meanwhile, physicochemical characterization of the solid substrate biomass, including particle size and chemical composition, also apparently affect the SSF performance [17,18].

**Table 1:** Summary of literature reports on solid state fermentation (SSF) of sweet sorghum stalk (SSS) for bioethanol production

**Feedstock**	**Particle size**	**Moisture content (w/w) (%)**	**Fermentation temperature (°C)**	**Fermentation time (h)**	**Strain**	**Ethanol yield**	**Reference**
Sweet sorghum stalk	2.0 cm long, 0.15 cm thickness	70	35	28	*Saccharomyces cerevisiae*	22.7 g ethanol/100 g SSS (DM)	This study
Sweet sorghum stem	1 to 2 mm in diameter, 3 to 50 mm in length	70	28	30	*S. cerevisiae* TSH1	6.25 g ethanol/100 g drystalk	Li *et al*. [23]
Sweet sorghum stalk	2 mm	75	42	60	*Issatchenkia orientalis* IPE 100	25 g ethanol/100 g dry stalk	Kwon *et al*. [24]
Dry sweet sorghum stalk	0.9 to 1.6 mm	76.5	35 to 40	30	Angel active dry yeast	Y_ethanol/sugar_ 0.2593	Shen and Liu [25]
Sweet sorghum stalk	1.5 mm	75	37	50	*S.cerevisiae* AF37X	7.9 g ethanol/100 g fresh stalk	Yu *et al*. [9]

DM, dry matter; SSF, solid state fermentation; SSS, sweet sorghum stalk.

The present study aims to systematically identify the effects of gas stripping on SSF performance of SSS solid substrates for bioethanol production compared with TS-SSF. The effects of gas stripping on the ethanol yield and the heat and mass transfer efficiency during SSF were analyzed. Ethanol stripping efficiency was considered as the key metric for evaluation of the ethanol online separation efficiency and GS-SSF performance. Fermentation conditions that might affect GS-SSF performance (including initial gas stripping time, gas stripping temperature, fermentation time, and particle thickness) were also investigated in the present study. Meanwhile, the relations of the ethanol content distribution between the gas phase by gas stripping and fermentation of the solid substrate residue were established in GS-SSF. To our knowledge, this is the first systematic study on gas stripping enhancing SSF performance from SSS for bioethanol production without physicochemical pretreatment.

## Results and discussion

### Composition analysis of sweet sorghum stalk (SSS)

The chemical composition of feedstock is crucial for the biomass conversion process, especially for SSF from solid substrates [1,17,18]. The chemical composition analysis of SSS is shown in Table 2 and expressed in total matter (TM) and dry matter (DM). The results showed that the moisture content of SSS, which is one of the most important parameters in SSF [17,21], reached 76.2% based on TM. Previous studies confirmed that high moisture content resulted in the decrease of solid substrate biomass porosity, which in turn affected the gas transfer and exchange efficiency during SSF. On the other hand, low moisture content may lead to poor accessibility of nutrients, resulting in poor microbial growth and fermentation performance [17,27]. Low substrate moisture content also resulted in secondary metabolite formation due to the difficulty of solute diffusion to the cells of microorganisms during SSF [28]. The moisture content used in this study was maintained at 70% based on the experimental optimum of our previous studies. SSS contained plentiful soluble sugars (43.07% sucrose, 7.69% glucose, and 4.71% fructose based on DM), which were easily directly converted to ethanol by microbial fermentation. The total soluble sugars of SSS were obviously higher than that of the literature report [9,14,23], which may reflect a difference in SSS itself. The content of crude fiber, which mainly contains cellulose, hemicellulose, and lignin, was 36.34% (DM). Thus, the total carbohydrate content of SSS was apparently higher than that of other agricultural straws [4,29]. Previous studies reported that the bioconversion efficiency of biomass is positively correlated with the amount of salts generated from the plant cell [30,31]. The ash content of SSS reached 5.13% (DM) in the present study. It should provide inorganic salt for microbial growth which may improve microbial activity, and hence enhance microbial fermentation performance. Therefore, the results implied that SSS should be a suitable biomass for the bioconversion process of bioethanol production.

**Table 2:** Chemical components of sweet sorghum stalk (SSS) based on total matter (TM) and dry matter (DM)

**Component**	**TM(g/100 g)**	**DM(g/100 g)**
Sucrose	10.25 (0.25)	43.07 (1.21)
Glucose	1.83 (0.09)	7.69 (0.88)
Fructose	1.12 (0.06)	4.71 (0.92)
Crude fiber	8.65 (0.39)	36.34 (1.56)
Ash	1.22 (0.22)	5.13 (0.71)
Others	0.73 (0.22)	3.07 (0.83)
Moisture	76.2 (2.1)	-

Standard deviations are shown in parentheses. SSS, sweet sorghum stalk; TM, total matter; DM, dry matter.

### Determination of initial gas stripping time

**Figure 1:**
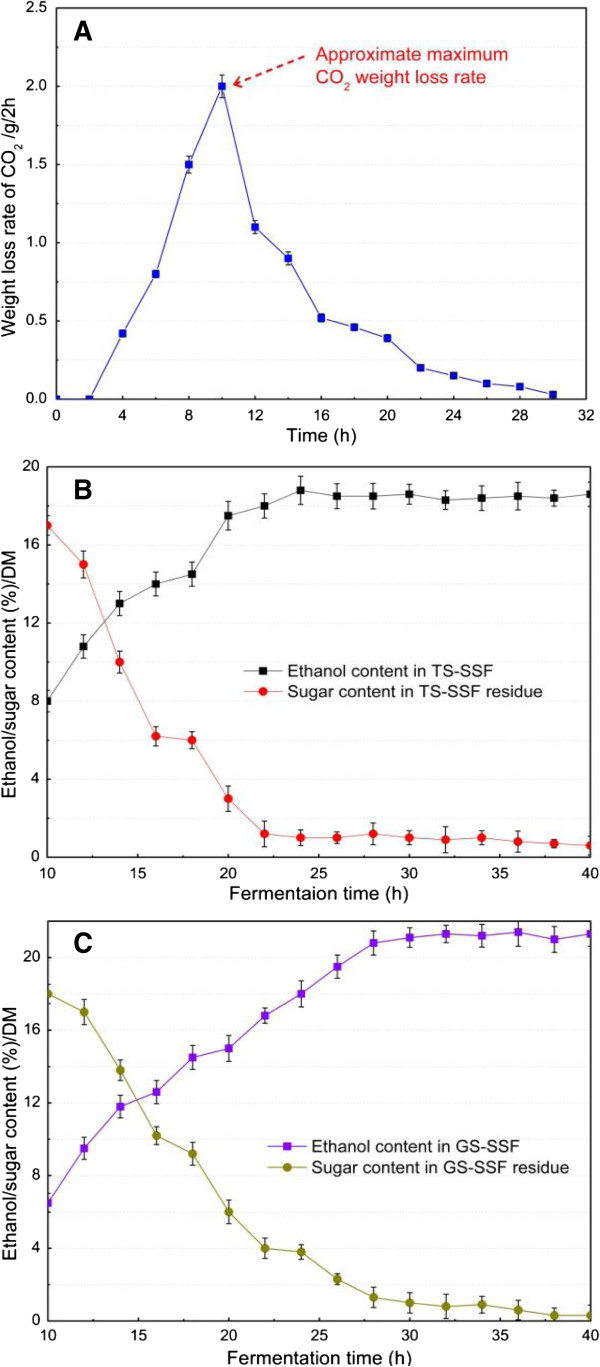
**Optimization of initial gas stripping time by CO**_**2 **_**weight loss rate and fermentation dynamics for ethanol production during TS-SSF and GS-SSF. (A)** CO_2_ weight loss rate. **(B)** Ethanol/sugar content in TS-SSF. **(C)** Ethanol/sugar content in GS-SSF. Fermentation conditions for determination of CO_2_ weight loss rate **(A)**: 70% moisture content, pH 5.0, 35°C, 0.5 g yeast/100 g SSS (DM), 2.0 cm long, and 0.15 cm thickness of particle. CO_2_ weight loss rate is defined as CO_2_ weight loss per 2 h. TS-SSF conditions **(B)**: 70% moisture content, pH 5.0, 35°C, 0.5 g yeast/100 g SSS (DM), 2.0 cm long, and 0.15 cm thickness of particle. GS-SSF conditions **(C)**: 70% moisture content, pH 5.0, 35°C, 0.5 g yeast/100 g SSS (DM), 2.0 cm long, and 0.15 cm thickness of particle; initial gas stripping time 10 h. DM, dry matter; GS-SSF, gas stripping solid state fermentation; SSS, sweet sorghum stalk; TS-SSF, traditional static solid state fermentation.

In the present study, the evaluation of initial gas stripping time during GS-SSF for conversion of SSS into bioethanol was carried out in shake flasks. Soluble sugars in SSS were converted to CO_2_ and ethanol by *Saccharomyces cerevisiae*. Due to the fact that CO_2_ is the other product of the SSS conversion process for ethanol production, the ethanol production rate could be directly described by the CO_2_ weight loss rate [32,33]. Therefore, the CO_2_ weight loss rate should be considered as the optimization objective of conversion biomass for bioethanol fermentation, which was also used as the indication of microbial activity and fermentation performance. Figure 1A shows that the CO_2_ weight loss rate obviously increased with the fermentation time from 0 h to 10 h, but then decreased with the fermentation time from 10 h to 30 h. The approximate maximum CO_2_ weight loss rate of 2.0 g/2 h was obtained at 10 h after inoculation during SSF, which indicated that the corresponding highest yeast activity and ethanol production rate should be obtained under this fermentation time. The results also implied that ethanol content in solid substrate should rapidly increase after 10 h of fermentation during SSF. The bioconversion process for bioethanol production from biomass is a typical end-product feedback inhibition process. In other words, high ethanol content reduces the fermentative activity of yeast, and hence reduces the ethanol yield and the conversion performance [26,34]. Due to the high end-product content in solid substrate biomass during SSF, the ethanol feedback inhibition effect on yeast cells should become more significant. Thus, excess accumulation of ethanol in the solid substrate should not be permitted for the ethanol production process and the ethanol content must be very highly controlled. In the present study, gas stripping was used for online separation of ethanol from the fermentation of solid substrate residue and removing the ethanol inhibition effect during SSF. The initial gas stripping time during SSF should be the balance of the capital cost of the stripping operation and the ethanol yield or the ethanol stripping efficiency. According to the above analysis, the optimal initial gas stripping time was determined as 10 h after inoculation during SSF.

### Optimization of ethanol fermentation time

The capital cost of biomass conversion for ethanol production is closely related to fermentation time [35], which should also obviously affect the gas stripping efficiency. Figure 1B and Figure 1C show the fermentation dynamics of TS-SSF and GS-SSF, respectively, for ethanol production from SSS. The results indicated that the ethanol content based on the DM of the solid substrate is a function of fermentation time. Ethanol content rapidly increased and sugar content obviously decreased with the fermentation time from 10 h to 24 h for TS-SSF and from 10 h to 28 h for GS-SSF after inoculation, respectively, but ethanol content then hardly increased for both TS-SSF and GS-SSF with the increase of fermentation time. This was due to the fact that the nutrition was affluent and easily obtained and utilized by yeast at the beginning of fermentation, while the available sugar content in the fermentation of solid substrate residue was low at the late stage of fermentation. The results showed that the fermentation time of the achievement of the highest ethanol content during TS-SSF was less than that of GS-SSF. It was also interesting to note that the sugar consumption rate and ethanol production rate were both lower for TS-SSF compared with that for GS-SSF. However, the ethanol content after 24 h of fermentation exhibited an opposite trend. From the fermentation point of view, the logical approach should be the selection of optimal fermentation time, leading to maximal ethanol yield based on economic analysis. To our knowledge, an increase of fermentation time obviously increases the capital cost of the bioethanol production process and the possibility of contaminative microbes. Meanwhile, the extension of fermentation time also increased the gas stripping operation cost during GS-SSF. High ethanol productivity and short fermentation time are needed, and it should be not a contradiction in ethanol production. The short-term fermentation may improve the ethanol yield due to the avoidance of end-product inhibition and ethanol volatilization and the reduction of contamination risk during SSF [22,36]. It can also improve the utilization efficiency of equipment, and hence reduce the process capital cost. Thus, the optimal ethanol fermentation time was determined as 28 h during SSF of SSS in the present study.

### Effect of temperature on online ethanol separation by gas stripping

**Figure 2:**
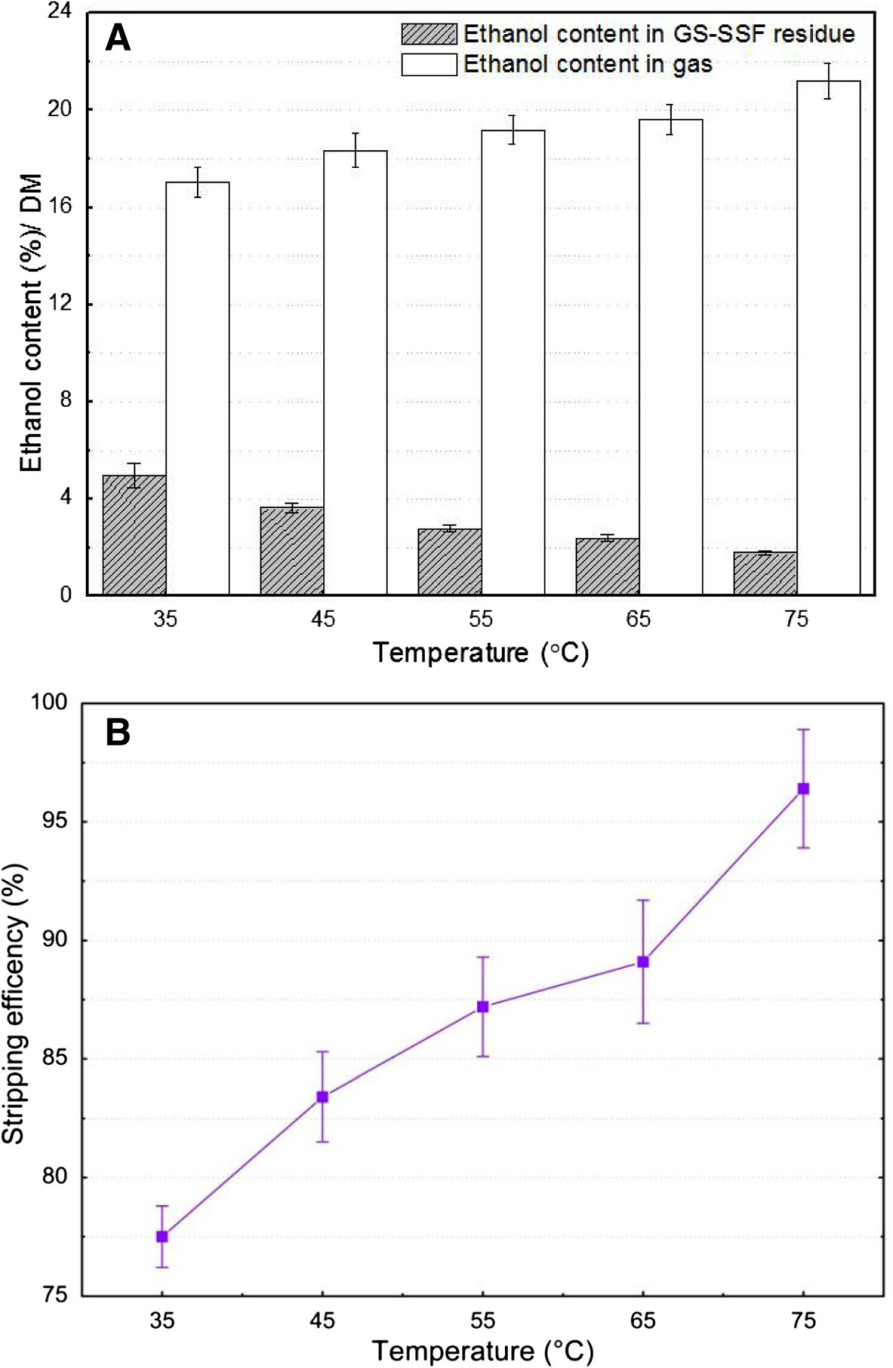
**Effect of temperature on ethanol distribution and ethanol stripping efficiency in the gas stripping experiment. (A)** Ethanol content. **(B)** Stripping efficiency. Gas stripping conditions: 70% moisture content, pH 5.0, 22 g ethanol/100 g SSS (DM), 2.0 cm long, and 0.15 cm thickness of particle. DM, dry matter; GS-SSF, gas stripping solid state fermentation; SSS, sweet sorghum stalk.

Temperature is one of most important parameters for fermentation and ethanol separation by gas stripping in the biomass conversion process [34,37]. Gas stripping experiments at different temperatures including 35, 45, 55, 65, and 75°C were performed with an initial ethanol content of 22% (w/w) in SSS solid substrate (Figure 2). The results clearly showed the importance of gas stripping temperature for ethanol distribution and ethanol stripping efficiency. With the increase of temperature, the ethanol content in solid substrate residue apparently decreased from 5.1% to 1.8%, while the ethanol content in the gas phase by gas stripping obviously increased from 16.9% to 20.1% (Figure 2A). The ethanol stripping efficiency increased by approximately 20% with temperature increasing from 35°C to 75°C, and the highest ethanol stripping efficiency (96.4%) was obtained at 75°C (Figure 2B). Increased temperature obviously improved the ethanol stripping efficiency because the partial pressure of ethanol in the gas phase and the ethanol diffusion rate in the pores of the solid substrate apparently increased. However, high ambient temperature increases the energy consumption and should reduce the energy utilization efficiency. Previous studies confirmed that the temperature of the solid substrate was also very critical during SSF, since it ultimately affected the activity of enzymes, growth of microbes, morphological characteristics of the solid substrate, formation of product, and mass and heat transfer [18,38,39]. The high temperature was helpful to online ethanol separation by gas striping, but it was harmful to yeast activity, especially when the temperature was more than 30°C, which was a contradiction during GS-SSF. In order to improve the ethanol stripping efficiency and strengthen the SSS conversion performance, thermal-tolerant alcohol active yeast were used for ethanol production during SSF in the present study. However, the fermentation temperature should be no more than 40°C. Therefore, considering all the above discussions, the optimum temperature for GS-SSF was determined as 35°C.

### Effect of particle thickness on GS-SSF

**Figure 3:**
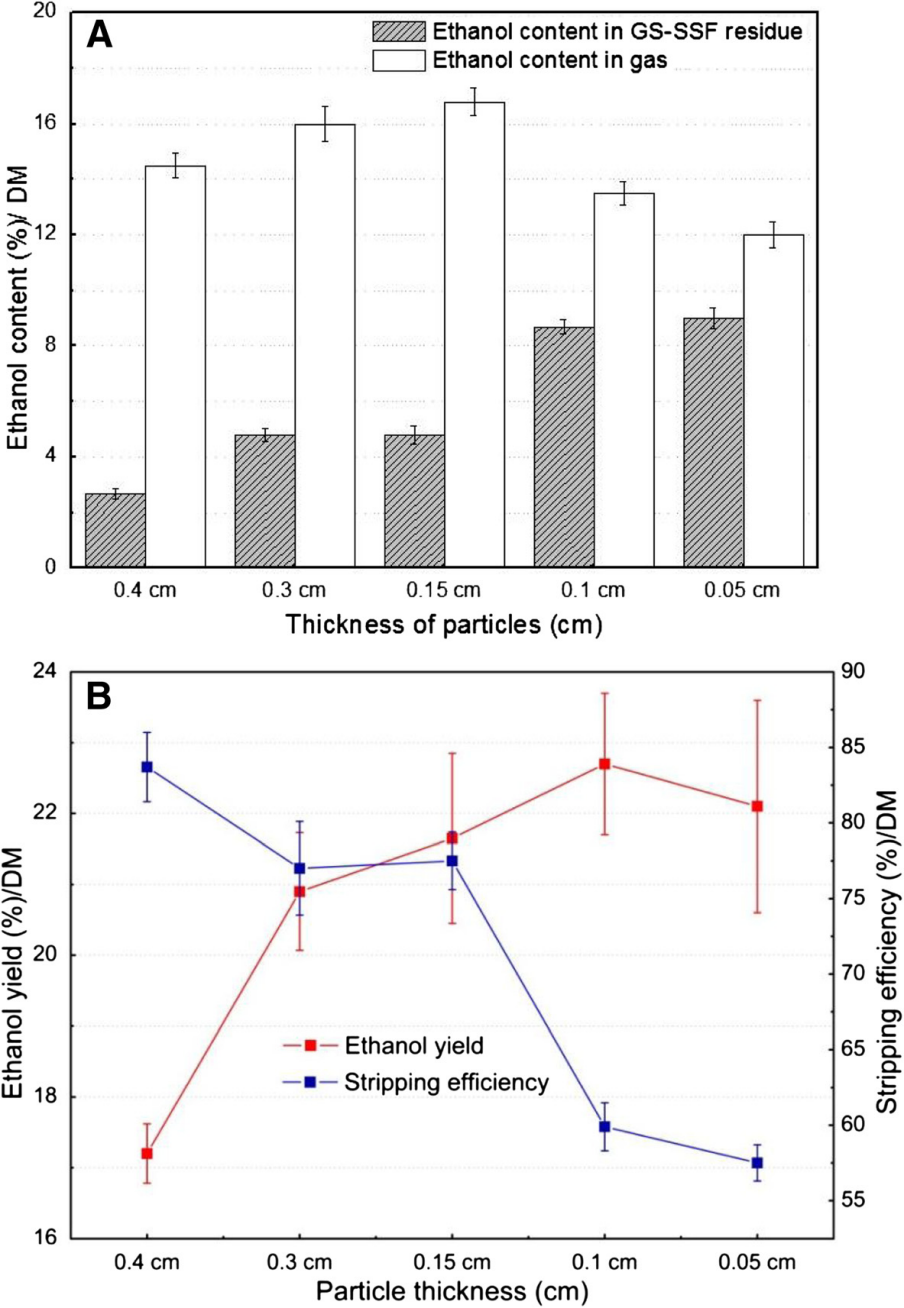
**Effect of particle thickness on the ethanol distribution, ethanol stripping efficiency, and ethanol yield during GS-SSF. (A)** Ethanol content. **(B)** Ethanol yield and stripping efficiency. Fermentation conditions: 70% moisture content, pH 5.0, 35°C, and 0.5 g yeast/100 g SSS (DM); initial stripping time 10 h and fermentation time 28 h. DM, dry matter; GS-SSF, gas stripping solid state fermentation; SSS, sweet sorghum stalk.

Different SSS particles, including 2.0 cm long and 0.4 cm, 0.3 cm, 0.15 cm, 0.1 cm and 0.05 cm thickness, were used as solid substrate and evaluated for improving the conversion performance during GS-SSF. The effect of particle thickness on the ethanol distribution, ethanol stripping efficiency, and ethanol yield of GS-SSF were systematically investigated and the results are given in Figure 3. The results showed that the thickness of particles obviously affected the ethanol distribution and ethanol stripping efficiency during GS-SSF. The ethanol content in solid substrate residues increased by about 3.6 times with the decrease of particle thickness from 0.4 cm to 0.05 cm, which meant that the smallest particle thickness within the present particle thickness range retained the highest ethanol content during GS-SSF (Figure 3A). The possible reason for this result was that the porosity of SSS greatly decreased with the decrease of SSS particle thickness, which reduced the gas diffusion rate in the pores of the SSS pile. Furthermore, the smaller particle thickness formed more sealed chambers, which may prevent the overflow of ethanol from the solid substrate. The ethanol content in the gas phase by gas stripping increased by 16% with the decrease of particle thickness from 0.4 cm to 0.15 cm, but it then obviously decreased by 40% with the decrease of particle thickness from 0.15 cm to 0.05 cm. The highest ethanol content in the gas phase by gas stripping was about 17% at 0.15 cm particle thickness (Figure 3A). These results confirmed the above discussions of the ethanol content distribution in solid substrate residues at different particle thicknesses.

Among the several parameters, particle size, which is closely related with microbial growth and activity and the fermentation performance, is a critical factor in SSF [38,40]. It was interesting to note that the ethanol yield apparently increased from 17% to 22% with particle thicknesses from 0.4 cm to 0.05 cm (Figure 3B). The maximum ethanol yield was obtained at the smallest particle thickness within the present particle thickness range. The reason for this phenomenon was that the particle size obviously affects the specific surface area of the solid substrate [41]. The available surface area for microbes increased with the decrease of particle thickness. In other words, the smaller particle thickness would provide a larger surface area for microbial attack. Due to the fact that a sufficient surface area was available for adequate nutrient diffusion and transfer, the growth rate of *S. cerevisiae* increased, and hence the ethanol production efficiency was improved. However, the energy consumption of size reduction of biomass is higher for small particle thickness than that for large thickness. In addition, too small particle thickness may result in the adhesion and agglomeration of the solid substrate during SSF, which may reduce the solid substrate porosity and the gas diffusion rate and increase the accumulation of heat. The reduced mass and heat transfer efficiency led to the poor growth of microbes, and hence the low product yield [22,40]. Therefore, it would be necessary to choose a suitable particle size for the biomass conversion process.

The ethanol stripping efficiency obviously decreased from 83.7% to 57.5% with particle thicknesses from 0.4 cm to 0.05 cm (Figure 3B). The maximum ethanol stripping efficiency (83.7%) was obtained at the largest particle thickness (0.4 cm). The possible reason for this result was that the particle thickness obviously affected the packing density of the biomass solid substrate and gas exchange efficiency during SSF [18,41]. The packing density which determined the solid substrate porosity and the solid substrate bed thickness decreased with the increase of particle thickness. The smallest packing density and the highest porosity of the SSS solid substrate pile were obtained at 0.4 cm thickness. Within the present particle thickness range, the larger particle thickness provided limited surface for microbial attack compared with the smaller thickness, but it provided larger inter-particle space of the solid substrate pile. The larger inter-particle space was helpful for gas diffusion and exchange during gas stripping, and hence improved the ethanol stripping efficiency.

The results implied that the highest ethanol yield was obtained at the smallest particle thickness, but the highest ethanol stripping efficiency was obtained at the largest particle thickness. High ethanol yield was required for the bioconversion process from biomass, while stripping efficiency was a key standard for the downstream process of product separation due to the fact that the high stripping efficiency reduced the capital cost in the industrial process. As a result, the analysis of the ethanol yield and ethanol stripping efficiency combined with energy consumption of size reduction suggested that the 2.0 cm long and 0.15 cm particle thickness was more suitable for the SSS bioconversion process by GS-SSF.

### Traditional static solid state fermentation (TS-SSF) versus gas stripping solid state fermentation (GS-SSF)

**Figure 4:**
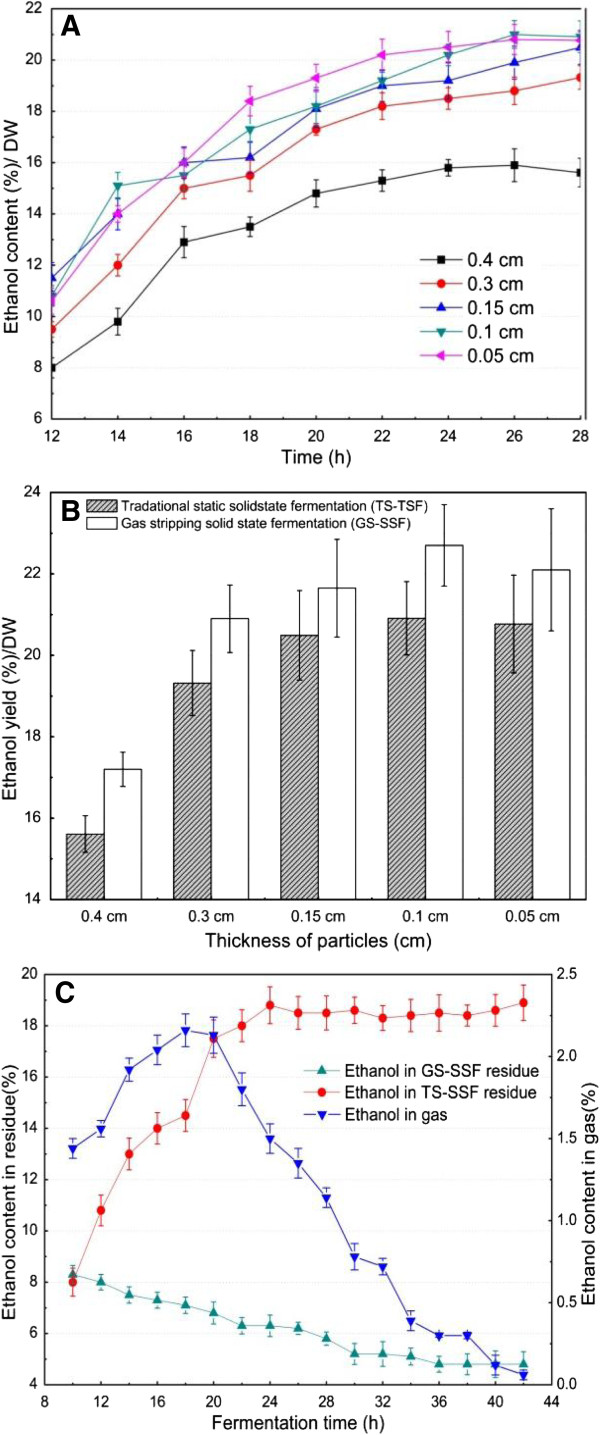
**TS-SSF and GS-SSF for bioethanol production from sweet sorghum stalk (SSS) at different particle thicknesses. (A)** Fermentation kinetic profile of TS-SSF for bioethanol production at different SSS particle thicknesses. **(B)** Ethanol yield for TS-SSF and GS-SSF at different SSS particle thicknesses. **(C)** Relations between the remaining ethanol in the solid substrate residue and gas stripping ethanol in the gas phase during GS-SSF. TS-SSF conditions: 70% moisture content, pH 5.0, 35°C, and 0.5 g yeast/100 g SSS (DM); fermentation time 28 h. GS-SSF conditions: 70% moisture content, pH 5.0, 35°C, and 0.5 g yeast/100 g SSS (DM); initial gas stripping time 10 h and fermentation time 28 h. DM, dry matter; GS-SSF, gas stripping solid state fermentation; SSS, sweet sorghum stalk; TS-SSF, traditional static solid state fermentation.

The effect of particle thickness on kinetic parameters of TS-SSF from SSS for ethanol production are shown in Figure 4A. The results showed that the TS-SSF performance was strongly influenced by fermentation time and particle thickness. The ethanol content in the solid substrate following the typical batch static fermentation pattern rapidly increased with fermentation time from 10 h to 20 h, and then the ethanol production rate slowed down for all particle thicknesses. One reason for this phenomenon was that the initial ethanol production rate was a function of the accessible surface area for microbes, while the slowdown of the ethanol production rate at the later stage of fermentation was due to the low nutrient content in the solid substrate and the difficulty of attacking the remaining nutrients. Another important reason for this result was that ethanol production from solid substrate biomass was an end-product feedback inhibition reaction. The high ethanol content in the solid substrate at the later stage of TS-SSF should result in a significant ethanol inhibition effect on yeast cells, which obviously reduced the ethanol productivity. When fermentation time varied from 10 h to 20 h, the ethanol content obviously increased with a particle thickness decrease from 0.4 cm to 0.05 cm. Furthermore, the smaller particle thickness also obtained higher ethanol content at the later stage of TS-SSF compared with the larger thickness. These trends were approximately similar to the GS-SSF process at different SSS particle thicknesses.

The ethanol yields during TS-SSF and GS-SSF at different particle thicknesses were compared in the present study (Figure 4B). The results showed that the ethanol yield was higher during GS-SSF than TS-SSF under the same particle thickness, and the corresponding ethanol yield increased by 6% to 10% for GS-SSF compared with TS-SSF. Previous studies reported that the mass and heat transfer was a major bottle neck for SSF, and especially for large-scale production [17,18,22]. During SSF, a large amount of heat was generated and the temperature gradient was formed in the solid substrate, which was directly related to the metabolic activities of the microorganisms. The solid substrate biomass used for SSF has low thermal conductivity, and hence the removal of heat from the inner solid substrate residue could be very slow and inefficient [17,22]. Worse still, accumulation of heat was often fatal during the fermentation process because the increased temperature could affect the growth of microorganisms and the product formation. Meanwhile, the main mass transfer method in the solid substrate is molecular diffusion during TS-SSF, which obviously affects the nutrition transfer efficiency. To remove the accumulated heat and the temperature gradient in the solid substrate and strengthen the fermentation performance, aeration was introduced into the SSF process in previous studies [21,42]. The application of gas stripping to separate ethanol under a certain temperature removed the heat generated by microbial metabolism through the strengthened gaseous phase during SSF, which improved the mass and heat transfer efficiency. Meanwhile, the gas stripping operation also provided agitation and loosened the solid substrate bed, which was not only helpful for the growth of microbes but also improved the mass and heat transfer efficiency through strengthening the solid phase during GS-SSF.

On the other hand, the ethanol content in SSF was much higher than that in SmF [21,34,35]. Thus, the ethanol inhibition effect on yeast becomes significant during SSF. Figure 4C shows that the ethanol content in the solid substrate residue during TS-SSF rapidly increased with fermentation time from 10 h to 28 h, and it then reached about 18% and kept at a high level at the later stage of fermentation. However, the ethanol content in the solid substrate was apparently less than 8% at 10 h and decreased from 8% to 5% with fermentation time from 10 h to 42 h after gas stripping during GS-SSF, which was obviously lower than that during TS-SSF. The results also indicated that the ethanol content per 2 h in the gas phase by gas stripping increased with fermentation time from 10 h to 20 h, and then decreased with the progress of fermentation during GS-SSF. The ethanol gas stripping rate was higher than 1.0% (w/w) per 2 h with the fermentation time from 10 h to 28 h. These results revealed that gas stripping significantly carried off the ethanol from fermentation of the solid substrate residue during GS-SSF, which obviously removed the ethanol inhibition effect. The metabolic activities of yeast were improved, leading to the increase of cell density. As a result, the solid substrate was fully utilized by yeast and the ethanol yield was apparently improved during GS-SSF compared with TS-SSF. Meanwhile, gas stripping may also result in an increase in ethanol productivity due to the reduced by-product formation by yeast metabolism during GS-SSF.

**Figure 5:**
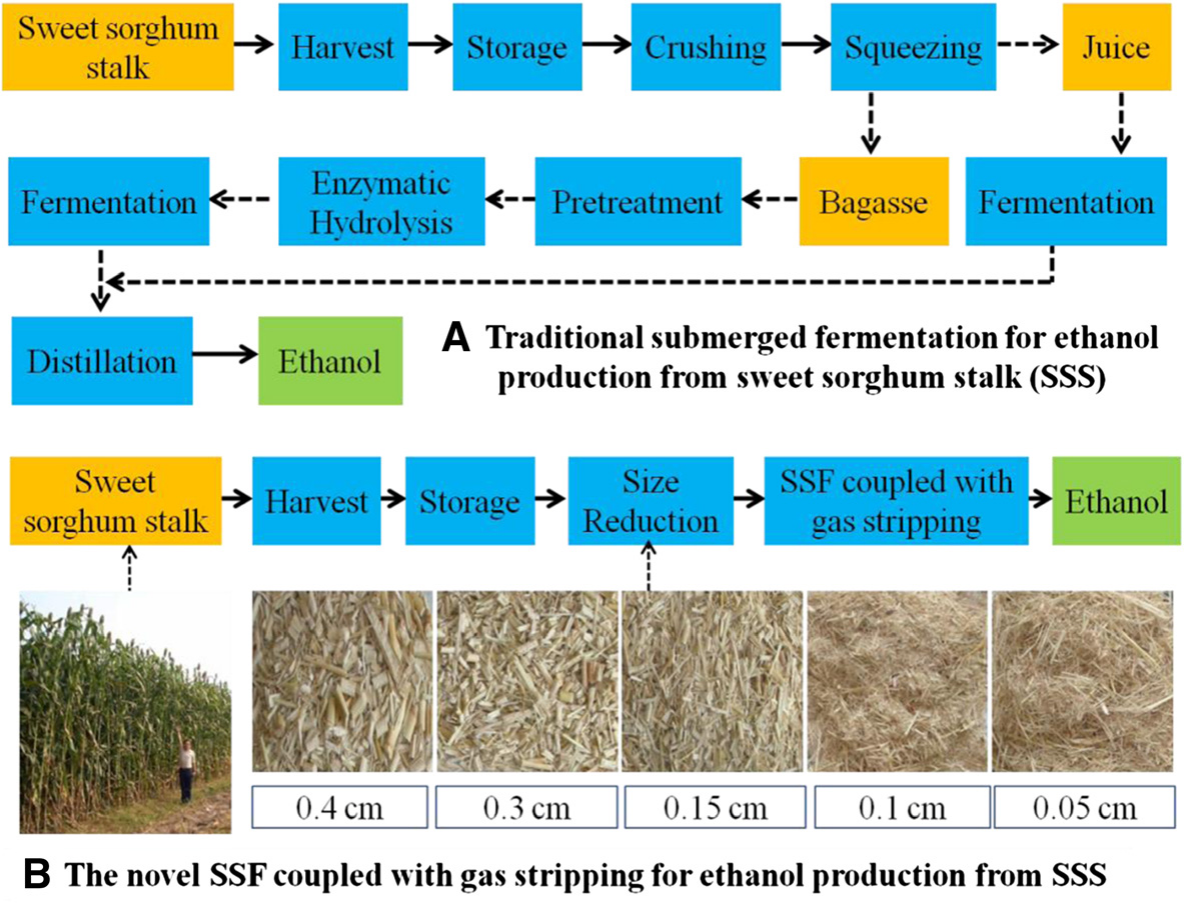
**Process diagram of bioconversion process for bioethanol production from sweet sorghum stalk (SSS). (A)** Traditional SmF process for bioethanol production from SSS. **(B)** The novel SSF coupled with gas stripping process for bioethanol production from SSS. SmF, submerged fermentation; SSF, solid state fermentation; SSS, sweet sorghum stalk.

**Figure 6:**
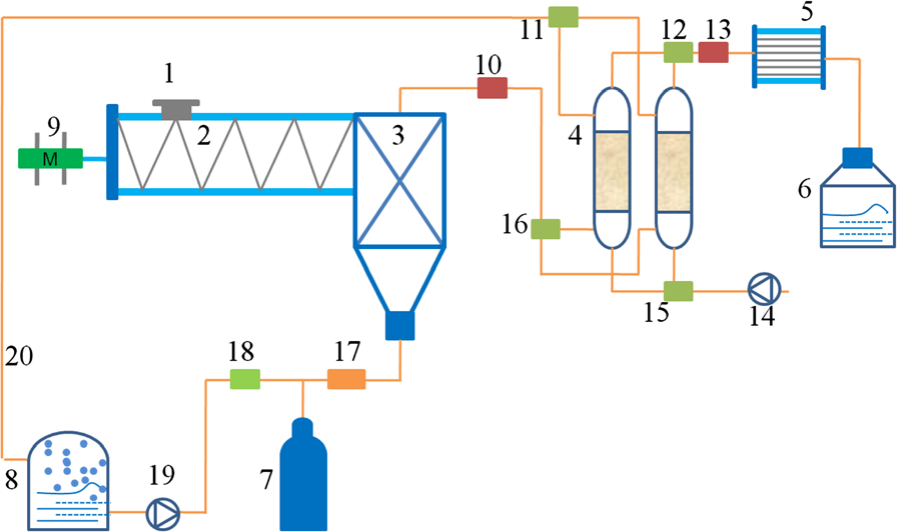
**Schematic of the novel GS-SSF reactor system for bioethanol production by continuous fermentation and online ethanol separation from sweet sorghum stalk (SSS).** 1, Air seal machinery; 2, fermenter; 3, gas-stripping tank; 4, carbon adsorption column; 5, condensator; 6, receptacle; 7, CO_2_ gas bottle; 8, gasholder; 9, stepping motor; 10, first alcohol concentration gauge; 11, first three-way valve; 12, first valve; 13, second alcohol concentration gauge; 14, air pump; 15, second three-way valve; 16, second valve; 17, mass flow controller; 18, third valve; 19, compression pump; and 20, pipeline. GS-SSF, gas stripping solid state fermentation; SSS, sweet sorghum stalk.

Although the bioconversion processes for bioethanol production from SSS were simplified compared with the traditional SmF process (Figure 5), TS-SSF was in accordance with the traditional method of intermittent operation. The products and the solid substrate residues should be transferred into another reactor and the distillation procedures should also be conducted after TS-SSF. These processes are very tedious and will definitely take a very long time, which obviously increased the product recovery costs during the bioethanol production process. Thus, the TS-SSF process does not meet the requirements of the low time utilization ratio and the high yield and efficiency of industrial production. In the present research, continuous operation for bioconversion of SSS biomass was achieved due to the coupling of the ethanol fermentation and the online ethanol separation through GS-SSF. During GS-SSF, the fermented mixtures were felled into the gas-stripping tank automatically because the fermenter and gas-stripping tank were linked vertically and sealed as a whole (Figure 6). From solid substrate preparation to ethanol separation, all operations were completed in the novel GS-SSF reactor, which avoided the influence of the external environment. The GS-SSF process eliminated the need for separate reactors and further simplified the bioconversion process. It also saved production time and labor and improved the product yield, and hence reduced the capital cost of the bioethanol production process.

Therefore, gas stripping strongly enhanced the SSF performance due to the removal of the ethanol inhibition effect, strengthening of mass and heat transfer, and simplification of the production process compared with TS-SSF. In the present study, SSF coupled with gas stripping (GS-SSF) was a novel SSS bioconversion process for bioethanol production compared with TS-SSF, especially compared with the traditional SmF process (Figure 5).

## Conclusions

Our results showed that gas stripping obviously enhanced GS-SSF performance of SSS compared with TS-SSF. Optimal GS-SSF conditions of initial gas stripping time, gas stripping temperature, fermentation time, and particle thickness were 10 h, 35°C, 28 h, and 0.15 cm, respectively. Gas stripping apparently improved the ethanol yield because the ethanol inhibition effect was removed and the mass and heat transfer efficiency improved. Meanwhile, GS-SSF eliminated the need for separate reactors and further simplified the ethanol production process. Therefore, SSF of SSS coupled with gas stripping would be more suitable to improve fermentation ability, and hence reduce the capital cost of the bioconversion process compared with TS-SSF.

## Materials and methods

### The conversion process diagram and the novel GS-SSF reactor system

The conversion process diagram of a novel SSF coupled with gas stripping for bioethanol production from SSS at different particle thicknesses is given in Figure 5, compared with traditional bioethanol production by SmF.

The novel GS-SSF reactor system is illustrated in Figure 6. It mainly consists of a fermenter, a gas-stripping tank, two carbon adsorption columns, a condensator, a CO_2_ gas bottle, and a stepping motor.

### Raw material preparation

The SSS used in the present study was harvested from the suburb of Beijing, China. For composition analysis, raw material was air-dried to the moisture content of 5% to 10%, and then milled by knife mill (MQF-420, BJZKRF, Beijing, China). The milled raw material was passed through a screen of 2 mm and stored in sealed bags at 4°C. For SSF, raw materials were first cut into 2.0 cm long pieces by knife mill, and then torn into 0.4 cm, 0.3 cm, 0.15 cm, 0.1 cm, and 0.05 cm thicknesses by tearing chopper (Y-S800, BJZKRF), respectively.

### Microorganism and seed culture preparation

The *S. cerevisiae* used in this study was obtained from Hubei Angel Yeast Co., Ltd (Hubei, China). *S. cerevisiae* was pre-cultivated in YPD medium (20 g/L glucose, 10 g/L yeast extract, and 20 g/L peptone) at 30°C and 200 rpm for 15 h. The cells were then inoculated to secondary seed liquid culture medium consisting of 20 g/L glucose, 10 g/L yeast extract, and 20 g/L peptone, and were cultivated at 30°C and 200 rpm for 15 h. The initial optical density (OD) at 600 nm for secondary seed was 0.05.

### TS-SSF

Five different biomass particles were placed in 1.0 L shake flasks capped with rubber stoppers perforated with a syringe needle for gas release. Next, 0.5 g ammonium sulfate/100 g SSS (DM) and 0.5 g calcium chloride/100 g SSS (DM) were added and mixed evenly. The moisture content of SSS was adjusted to 70% by deionized water, and the initial pH was adjusted to 5.0. The mixture was then sterilized at 121°C for 15 min. After sterilization, 0.5 g yeast/100 g SSS (DM) was added into the mixture and mixed well, and the shake flask was then kept statically in the water bath at 35°C. The fermentation conditions of TS-SSF were based on the experimental optimum of our previous studies.

### GS-SSF

For GS-SSF, 0.5 g ammonium sulfate/100 g SSS (DM) and 0.5 g calcium chloride/100 g SSS (DM) were added into five different biomass particles and mixed well. The moisture content of SSS was adjusted to 70% by deionized water, and the initial pH was adjusted to 5.0. After heat sterilization at 121°C for 15 min, the mixture was cooled and inoculums of 0.5 g yeast/100 g SSS (DM) were added into the mixture and blended well. As shown in Figure 6, the solid substrate mixtures after sterilization and inoculation were top-loaded into the fermenter through air seal machinery. During ethanol fermentation, the solid substrate mixtures were pushed from the fermenter towards the gas-stripping tank at a predetermined speed by stepping motor. When the solid substrate mixtures reached the gas-stripping tank, ethanol was gas-stripped by CO_2_ with 10 kg/h at different times. The mixtures of ethanol and CO_2_ went through an activated carbon adsorption column, and ethanol was absorbed. The absorbed ethanol was desorbed by heating the activated carbon adsorption column and recovered in the receptor by the condensator. The remaining CO_2_ was recycled, and it was saturated by the humidifier before it was injected into the gas-stripping tank again.

### Determination of CO_2_ weight loss

Release of CO_2_ during SSF was adopted to evaluate the fermentation power of ethanol production from SSS. The reaction equation for growth of *S. cerevisiae* on glucose is:

$$C_{6}H_{12}O_{6}\to 2C_{2}H_{5}\mathrm{OH}+2{\mathrm{CO}}_{2}$$

Carbohydrates were converted to CO_2_ and ethanol. Production of CO_2_ overflowed from the shake flask caused a decrease in the weight of the fermented substrate, hence the weight of the shake flask reduced. Weight loss of CO_2_ in fermentation was measured by an accurate balance (0.01 g). It can be measured based on the total weight loss of the shake flask every 2 hours. The CO_2_ weight loss rate was defined as CO_2_ weight loss per 2 h.

### Analytical methods and calculations

The composition analysis of SSS was conducted according to the Laboratory Analysis Protocol (LAP) of the National Renewable Energy Laboratory (NREL), Golden, CO, USA [43-45]. The moisture content of SSS was determined using oven drying at 105°C for 24 h. The concentrations of sugar and ethanol were analyzed by HPLC (Agilent 1200, Agilent Technologies, Santa Clara, CA, USA), equipped with a refractive index detector and an Aminex HPX-87H carbohydrate analysis column (Bio-Rad, Hercules, CA, USA) at 35°C with 5 mM H_2_SO_4_ as the mobile phase at a flow rate of 0.6 mL/min. Sucrose, glucose, fructose, and ethanol standards used in the experiment were analytical grade and were purchased from Sigma-Aldrich (St Louis, MO, USA).

The ethanol yield was used as the key metric for evaluation of SSF performance. Ethanol stripping efficiency was used for determination of the online ethanol separation efficiency in GS-SSF. They were calculated as follows:

(1) $$\begin{array}{l} \text{Ethanol}\;\text{yield}\;\left(\%\right)=\text{Ethanol}\;\text{content}\;\text{after}\;\mathrm{SSF}\\ \;/\text{Raw}\;\text{material}\;\text{based}\;\text{on}\;\text{dry}\;\text{matter}\\ \;\times \;100\% \end{array}$$

(2) $$\begin{array}{l} \text{Ethanol}\;\text{stripping}\;\text{effiency}\;\left(\%\right)\;=\;\text{Ethanol}\;\text{in}\;\text{gas}\;\text{phase}\\ \;/\left[\text{Ethanol}\;\text{in}\;\text{gas}\;\text{phase}\right)\\ \;\left(+\text{Ethanol}\;\text{in}\;\text{residue}\right]\\ \;\times \;100\% \end{array}$$

## Abbreviations

DM: Dry matter; GS-SSF: Gas stripping solid state fermentation; HPLC: High performance liquid chromatography; LAP: Laboratory Analysis Protocol; NREL: National Renewable Energy Laboratory; OD: Optical density; SmF: Submerged fermentation; SSF: Solid state fermentation; SSS: Sweet sorghum stalk; TM: Total matter; TS-SSF: Traditional static solid state fermentation; YPD: Yeast extract peptone dextrose.

## Competing interests

The authors declare that they have no competing interests.

## Authors’ contributions

CHZ and LZH participated in the design of the study. LZH performed the statistical analysis and drafted the manuscript. LZH and DSH carried out the SSF experiments. LZH revised the manuscript. All authors provided critical input to the manuscript and read and approved the final manuscript.
