# Erratum to: A novel solid state fermentation coupled with gas stripping enhancing the sweet sorghum stalk conversion performance for bioethanol

**DOI:** 10.1186/s13068-015-0365-1

**Published:** 2015-11-17

**Authors:** Hong-Zhang Chen, Zhi-Hua Liu, Shu-Hua Dai

**Affiliations:** State Key Laboratory of Biochemical Engineering, Institute of Process Engineering, Chinese Academy of Sciences, Beijing, 100190 China; Graduate University of Chinese Academy of Sciences, Beijing, 100190 China

## Erratum to: Biotechnology for Biofuels (2014) 7:53 DOI 10.1186/1754-6834-7-53

After publication, the authors noticed an accidental error existing in this article [1]. In Table 1, they mistakenly listed the Ethanol yield of sweet sorghum stem as “6.25 g ethanol/100 g drystalk”. This information should be corrected as “6.25 g ethanol/100 g fresh stalk” (Table 1). This change will in no manner affect the outcome/interpretation of the experiments as described in the original publication. The authors apologize for any inconvenience caused.

**Table 1:** Summary of literature reports on solid state fermentation (SSF) of sweet sorghum stalk (SSS) for bioethanol production

Feedstock	Particle size	Moisture content (w/w) (%)	Fermentation temperature (°C)	Fermentation time (h)	Strain	Ethanol yield	Reference
Sweet sorghum stalk	2.0 cm long, 0.15 cm thickness	70	35	28	*Saccharomyces cerevisiae*	22.7 g ethanol/100 g SSS (DM)	This study
Sweet sorghum stem	1 to 2 mm in diameter, 3 to 50 mm in length	70	28	30	*S. cerevisiae* TSH1	6.25 g ethanol/100 g fresh stalk	Li et al. [23]
Sweet sorghum stalk	2 mm	75	42	60	*Issatchenkia orientalis* IPE 100	25 g ethanol/100 g dry stalk	Kwon et al. [24]
Dry sweet sorghum stalk	0.9 to 1.6 mm	76.5	35–40	30	Angel active dry yeast	Y_ethanol/sugar_ 0.2593	Shen and Liu [25]
Sweet sorghum stalk	1.5 mm	75	37	50	*S. cerevisiae* AF37X	7.9 g ethanol/100 g fresh stalk	Yu et al. [9]

DM, dry matter; SSF, solid state fermentation; SSS, sweet sorghum stalk
